# The effect of repetitive transcranial magnetic stimulation on dorsolateral prefrontal glutamate in youth with treatment-resistant depression

**DOI:** 10.1186/1753-6561-9-S1-A9

**Published:** 2015-01-14

**Authors:** S Pradhan, A Kirton, G MacQueen, F MacMaster

**Affiliations:** 1 Royal College of Surgeons in Ireland, 123 St. Stephen’s Green, Dublin 2, Ireland; 2 Department of Psychiatry and Clinical Neurosciences, University of Calgary, 2500 University Dr. NW Calgary, Alberta, Canada, T2N 1N4

## Background

Major Depressive Disorder (MDD) is a debilitating psychiatric disorder characterized by feelings of low self-worth, loss of interest and suicidal thoughts. An estimated 350 million people are affected worldwide, 15% being adolescents. Repetitive transcranial magnetic stimulation (rTMS) is a non-invasive intervention that modulates cortical excitability by inducing electric currents in neurons by administering pulsating magnetic fields to the scalp. Studies have shown the left dorsolateral prefrontal cortex (DLPFC) to be implicated in positive effects on emotion, and glutamate/Glx levels to be decreased in MDD patients. In adults, rTMS has been shown to significantly improve mood, decrease Hamilton Depression Scores and increase glutamate, glutamine and choline levels in the DLPFC. We hypothesize an increase in DLPFC glutamate levels following treatment.

## Methods

Eleven treatment-resistant MDD patients (4 females and 7 males, ages 15-21) were recruited and clinically assessed using the Hamilton Depression/Anxiety Rating Scale and the Children’s Depression Rating Scale at baseline, and during each week of treatment for three weeks. Participants also underwent baseline and post-treatment MRI scans. Magnetic resonance spectroscopy was used to measure glutamate levels and data was analysed using the LC model method. TMS treatments were performed daily for three weeks and treatment response was defined as ≥50% decrease in baseline Ham-D rating scale.

## Results

In general, rTMS treatments were well tolerated, however some patients described minor side effects of mild headaches and scalp discomfort. Seven patients were treatment responders who showed symptom improvement, a 62% decrease in Ham-D scores from 25.43 (±7.85) to 9.57 (±1.51) and a 30% decrease in CDRS scores from 74.43 (±11.04) to 52.14 (±8.99). Ham-A scores decreased 78% from 23.86 (±9.65) to 5.29 (±3.55). In the left DLPFC, treatment responder glutamine levels increased by 5% and Glx levels increased by 10% in the DLPFC. Responders also showed lower baseline glutamate levels at 8.73 (±1.21) mmol/kg-wet-weight, which increased by 12% to 9.77 (±1.18) following treatment. Interestingly, non-responders had higher baseline glutamate levels at 11.87 (±0.47) and levels decreased by 9% to 10.75 (±0.13).

## Conclusions

These findings are consistent with previous literature. Evidence of increases in excitatory neurotransmitter levels in the DLPFC and alleviation of symptoms following treatment indicate that rTMS exerts anti-depressant effects and can be pursued as a safe and effective therapy for adolescent MDD.

